# Can CANVAS due to *RFC1* biallelic expansions present with pure ataxia?

**DOI:** 10.1136/jnnp-2023-331381

**Published:** 2023-07-06

**Authors:** Marios Hadjivassiliou, Riccardo Currò, Nick Beauchamp, Natalia Dominik, Richard A Grunewald, Priya Shanmugarajah, Panayiotis Zis, Nigel Hoggard, Andrea Cortese

**Affiliations:** 1 Academic Department of Neurosciences, Royal Hallamshire Hospital, Sheffield Teaching Hospitals NHS Trust and University of Sheffield, Sheffield, UK; 2 Department of Neuromuscular Disease, UCL Queen Square Institute of Neurology, London, UK; 3 Department of Brain and Bahavioural Sciences, University of Pavia, Pavia, Italy; 4 Sheffield Diagnostic Genetics Service, Sheffield Children's Hospital NHS Foundation Trust, Sheffield, UK; 5 Academic Department of Neurology, University of Cyprus, Nicosia, Cyprus; 6 Department of Infection, Immunity and Cardiovascular Disease, The University of Sheffield, Sheffield, UK

**Keywords:** CEREBELLAR ATAXIA, NEUROPATHY

## Abstract

**Background:**

Biallelic expansion of AAGGG in the replication factor complex subunit 1 (*RFC1*) was identified as a major cause of cerebellar ataxia, neuropathy (sensory ganglionopathy, or SG) and vestibular areflexia syndrome (CANVAS). We wanted to clarify if *RFC1* expansions can present with pure ataxia and if such expansions could be responsible for some cases where an alternative diagnosis had been made.

**Methods:**

We identified patients with a combination of ataxia and SG and no other cause found, patients where an alternative diagnosis had been made, and patients with pure ataxia. Testing for *RFC1* expansions was done using established methodology.

**Results:**

Among 54 patients with otherwise idiopathic sporadic ataxia without SG, none was found to have *RFC1* expansions. Among 38 patients with cerebellar ataxia and SG in which all other causes were excluded, 71% had *RFC1* expansions. Among 27 patients with cerebellar ataxia and SG diagnosed with coeliac disease or gluten sensitivity, 15% had *RFC1* expansions.

**Conclusions:**

Isolated cerebellar ataxia without SG makes the diagnosis of CANVAS due to *RFC1* expansions highly improbable, but CANVAS is frequently the cause of the combination of idiopathic cerebellar ataxia with SG. It is important to screen patients diagnosed with other causes of acquired ataxia and SG as a small percentage were found to have *RFC1* expansions.

## Introduction

In 2019, a biallelic expansion of AAGGG pentanucleotides in the second intron of the replication factor complex subunit 1 (*RFC1*) was identified as the main cause of cerebellar ataxia, neuropathy (sensory ganglionopathy, or SG) and vestibular areflexia syndrome (CANVAS).^1^ Subsequent studies reported a high prevalence of biallelic AAGGG expansions in cases with sporadic or familial (autosomal recessive) ataxia in which SG was recognised as a key feature of this syndrome.^2^ It has been shown that up to a third of cases diagnosed with idiopathic SG with or without ataxia and vestibular impairment carry *RFC1* expansions.^3^ SG is usually associated with sensory ataxia. Establishing any cerebellar involvement in the context of SG can be challenging in the absence of any cerebellar-related eye movement signs and without detailed imaging of the cerebellum (including magnetic resonance (MR) spectroscopy). Nonetheless, in all cases carrying *RFC1* expansions reported so far, SG seems to be an essential part of the syndrome. Our main aim in this study was to clarify if patients with *RFC1* expansions can present with pure ataxia in the absence of SG or any other form of peripheral nerve involvement. At the same time, we wanted to clarify if CANVAS could be responsible for some cases in which an alternative diagnosis for the ataxia and SG had already been made.

## Methods

### Patient selection and MR spectroscopy

The Sheffield Ataxia Centre cares for over 3000 patients with progressive ataxias. Genetic testing for common CAG repeat expansions and later through next-generation sequencing with a large panel of ataxia genes has been available through the Sheffield Diagnostic Genetics Service for a number of years. Access to the 100,000 Genomes Project and subsequently whole genome sequencing (WGS) under the National Health Service has enhanced our ability for better genetic characterisation.^4^ Despite these advanced genetic testing techniques, repeat expansions cannot always be reliably detected using such methodologies. In particular, identification of *RFC1* expansion from short-read WGS can be challenging given the large size of the expansion and its polymorphic content.

We identified patients with the typical phenotype of cerebellar ataxia and SG for testing for CANVAS. We do not routinely perform vestibular tests in our patients with progressive ataxia, although since the identification of CANVAS we have included the head thrust test as part of the standard bedside neurological examination of any patient with ataxia. We selected patients with sporadic idiopathic ataxia with no clinical or neurophysiological evidence of SG or any other peripheral nerve involvement to be also tested for CANVAS. These patients had extensive genetic testing already (using the techniques mentioned above) without any cause being identified. Finally, as the combination of cerebellar ataxia and SG can be a manifestation of gluten sensitivity/coeliac disease, we screened for CANVAS a cohort of patients diagnosed as having gluten-related neurological dysfunction who had ataxia and SG.

All patients provided written consent for genetic testing, which is standard practice for any genetic testing done at our institution. The genetic testing was done as part of the clinical care of the patients.

All patients attending the Sheffield Ataxia Centre routinely undergo MR spectroscopy of the cerebellum using a well-established methodology.^5^

### *RFC1* testing

The presence of *RFC1* expansions was assessed as previously described.^1^ Briefly, DNA was tested by flanking PCR and repeat-primed PCR (RPPCR) for AAGGG repeat expansions in *RFC1*. Samples without amplifiable products on flanking PCR and a positive RPPCR for the AAGGG repeat were tested by Southern blotting in order to confirm the presence and to measure the size of the biallelic *RFC1* expansions. Non-pathogenic AAAAG or AAAGG expansions were excluded by RPPCR in all the cases with positive RPPCR for AAGGG expansion.

### Southern blotting

Provided that enough DNA with good quality was available, samples were analysed by Southern blotting to confirm the presence and to measure the size of the expanded alleles. Five micrograms of DNA were digested with EcoRI (New England Biolabs) and ran for 16 hours on agarose gel. The separated DNA fragments were then transferred onto positively charged Nylon Membrane (Roche) using an upward transfer method. The membrane was hybridised with a sequence-specific digoxygenin (DIG)-labelled probe and incubated with an anti-DIG antibody (Roche). Finally, the membrane was exposed to a chemiluminescent substrate (CDP-Star, Roche) and DNA bands were visualised on an X-ray film. Bands were measured against DIG-labelled DNA Molecular Weight Marker II (Roche). A non-expanded allele generates a band of 5 kilobases (kb), while expanded alleles are observed in a range from 7 kb to 15 kb. Biallelic expansions of similar size are shown as a unique band.

## Results

**Figure 1:**
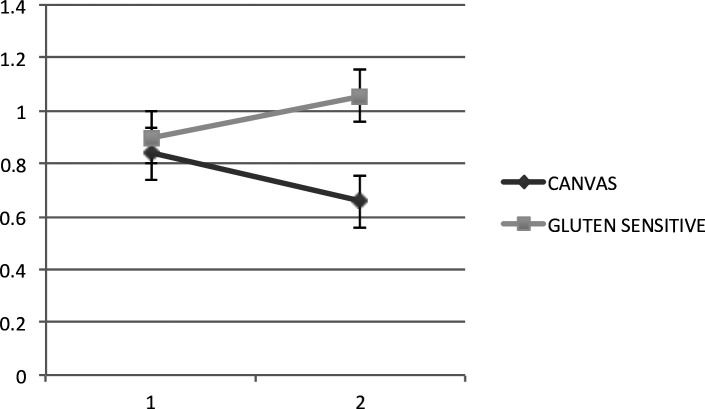
N-acetyl-aspartate to creatine (NAA:Cr) ratio (vertical axis) before and after the introduction of gluten-free diet (horizontal axis in years) using magnetic resonance spectroscopy of the cerebellum. The light grey line represents the change over time in eight patients with gluten sensitivity who went on a strict gluten-free diet, showing improvement in the NAA:Cr area ratio, associated with clinical improvement (mean NAA:Cr 0.8975, follow-up 1.055, p=0.0192). We have previously demonstrated a very good correlation between the NAA:Cr ratio and SARA score. The dark grey line represents the four patients with gluten sensitivity and a subsequent diagnosis of biallelic *RFC1* expansion, demonstrating that despite the gluten-free diet they continue to deteriorate (mean 0.8375, follow-up 0.6575, p=0.0216). CANVAS, cerebellar ataxia, neuropathy (sensory ganglionopathy) and vestibular areflexia syndrome; *RFC1*, replication factor complex subunit 1; SARA, Scale for the Assessment and Rating of Ataxia.

We tested 38 patients (from 38 families) attending the Sheffield Ataxia Centre who had both cerebellar ataxia and SG. All other possible causes for this combination had been excluded. Twenty-seven (71%) were found to have biallelic *RFC1* expansions in keeping with a diagnosis of CANVAS. Of these, only six had an autosomal recessive family history (one sibling affected). The rest were sporadic. We also tested 54 patients with otherwise idiopathic sporadic ataxia without any evidence of SG or any other type of peripheral neuropathy. None was found to carry *RFC1* expansion. Finally, we tested 27 patients with cerebellar ataxia and SG diagnosed with coeliac disease or gluten sensitivity. Four (15%) were positive for CANVAS. Table 1 summarises the prevalence of *RFC1* expansion among the three groups. Of interest is the fact that all 4 patients followed a progressive course despite strict gluten-free diet (GFD), while the remaining 23 patients responded well to GFD. Figure 1 illustrates the improvement on MR spectroscopy of the cerebellum for 8 out of the 23 patients who had repeat MR spectroscopy after strict GFD in contrast to the 4 patients with *RFC1* expansion where the MR spectroscopy worsened despite GFD. We have previously shown a very good correlation between MR spectroscopy and SARA score (Scale for the Assessment and Rating of Ataxia).^6^ The four patients who did not respond to GFD had positive serology for gluten sensitivity without having any enteropathy.

**Table 1:** Prevalence of *RFC1* expansion among the three patient groups

Patient groups	Prevalence of *RFC1* expansion, n/N (%)
Cerebellar ataxia and sensory ganglionopathy, no other cause found	27/38 (71)
Pure cerebellar ataxia without evidence of sensory ganglionopathy	0/54 (0)
Cerebellar ataxia and sensory ganglionopathy attributed to gluten sensitivity	4/27 (15)

*RFC1*, replication factor complex subunit 1.

## Discussion

CANVAS due to *RFC1* expansions is proving to be one of the most common autosomal recessive ataxias. In our large cohort of 3000 patients with ataxia, CANVAS due to *RFC1* expansions was the third most common autosomal recessive (AR) ataxia after Friedreich’s ataxia (FA; 28%) and spastic paraplegia type 7 (SPG7) (14%), accounting for 11% of all AR ataxias. Among patients with sporadic ataxia who were found to have a genetic defect, CANVAS accounted for 9%.^7^

There are specific characteristics that may allow the clinician to suspect CANVAS based on clinical history and examination: the presence of SG in combination with cerebellar ataxia and vestibular areflexia (positive head thrust test). Furthermore, patients with CANVAS often complain of a chronic dry cough that can sometimes precede the onset of neurological symptoms by many years. Chronic cough was certainly not something that the patients with pure ataxia were complaining of.

The current study demonstrates that in a patient with ataxia the absence of SG virtually excludes *RFC1* expansion as the cause of the ataxia. This finding is helpful in selecting patients for testing for *RFC1*, considering that intronic *RFC1* expansion test is not part of next generation sequencing (NGS) ataxia panels and its detection through WGS is not yet validated in a diagnostic setting.

From a clinical perspective, screening patients with the combination of cerebellar ataxia (the initial presenting complaint) and SG in the absence of any other cause has a diagnostic yield of 71% for *RFC1* expansion. This is significantly higher to what has been found when screening patients with idiopathic sensory neuropathy (34%), but similar (63%) to previously published study in the context of late-onset ataxia and sensory neuropathy.^1 3^ These observations emphasise the fact that CANVAS is indeed a syndrome that affects the cerebellum, the dorsal root ganglia and the vestibular system. By contrast, isolated cerebellar ataxia without SG is not likely to be caused by *RFC1* expansions, which means that a normal neurophysiology makes the diagnosis of CANVAS highly improbable. It is also of interest to consider published data showing 34% positivity for CANVAS in those patients with SG.^3^ Clinically, it is very difficult to exclude cerebellar involvement in these patients, particularly when they present and are followed up in neuromuscular clinics. In the absence of any eye signs suggestive of cerebellar involvement, these patients are unlikely to undergo brain imaging to look for cerebellar atrophy and even less likely to undergo MR spectroscopy of the cerebellum, which is probably the most sensitive tool of cerebellar involvement. Indeed, among the 34% of patients with CANVAS identified in a previous study, 70% complaint of unsteadiness when compared with 53% of the CANVAS-negative group. In addition, 50% of this cohort of patients recruited through neuromuscular clinics had some cerebellar signs on re-evaluation 10 years after disease onset.^3^ It is therefore very likely that patients with *RFC1* expansions presenting to neuromuscular clinics have clinical or subclinical involvement of the cerebellum at the time of presentation, although this is not routinely assessed.

The finding of 15% of patients with cerebellar ataxia and SG, who were thought to have gluten sensitivity as the cause, carrying biallelic *RFC1* expansions suggests that it is worth screening for such expansions, particularly if they seem to follow a progressive course and not respond to GFD. This is not just true for patients with gluten sensitivity but also for patients with Sjogren’s syndrome, another common cause of SG and ataxia.^8^ Indeed, cerebellar ataxia with SG has often been linked to autoimmune aetiology (paraneoplastic, gluten sensitivity, Sjogren’s syndrome).^9^

We have also screened for biallelic *RFC1* expansions in two patients prior to confirmation of paraneoplastic cerebellar ataxia and SG, both of whom were negative. Finally, the only other patient screened (negative) was a patient with ataxia and SG who proved to have late-onset FA. It is worth remembering that FA is also associated with cerebellar ataxia and SG and should be part of the differential of this combination, although contrary to CANVAS, FA presents at a much younger age and is associated with a number of other features (particularly when presenting early), allowing for a distinction between the two conditions.

In summary, the study confirms that biallelic *RFC1* expansions were a frequent (71%) cause of idiopathic cerebellar ataxia with SG. Importantly, we have shown for the first time that *RFC1* expansions are highly unlikely in cases with isolated cerebellar ataxia and in the absence of SG. Finally, we have also demonstrated that it is important to screen for *RFC1* expansions in patients with other suspected acquired causes of ataxia and SG as a small percentage (15%) of our cases previously diagnosed with gluten ataxia and unresponsive to GFD were found to carry biallelic *RFC1* expansions.
